# Raman Scattering Study on the Influence of E-Beam Bombardment on Si Electron Lens

**DOI:** 10.3390/molecules26092766

**Published:** 2021-05-08

**Authors:** Geon-Woo Lee, Young-Bok Lee, Dong-Hyun Baek, Jung-Gon Kim, Ho-Seob Kim

**Affiliations:** 1 Department of Physics and Nano-Science, Sunmoon University, Asan-si 31460, Korea; dlrjsdn0327@naver.com (G.-W.L.); polyhymnia@naver.com (Y.-B.L.); 2 Center for Next-Generation Semiconductor Technology, Sunmoon University, Asan-si 31460, Korea; dhbaek@sunmoon.ac.kr; 3 WaferMasters, Inc., Dublin, CA 94568, USA

**Keywords:** microcolumns, MEMS, silicon, carbon, contamination, Raman spectrum, e-beam, XPS

## Abstract

Microcolumns have a stacked structure composed of an electron emitter, electron lens (source lens), einzel lens, and a deflector manufactured using a micro electro-mechanical system process. The electrons emitted from the tungsten field emitter mostly pass through the aperture holes. However, other electrons fail to pass through because of collisions around the aperture hole. We used Raman scattering measurements and X-ray photoelectron spectroscopy analyses to investigate the influence of electron beam bombardment on a Si electron lens irradiated by acceleration voltages of 0, 20, and 30 keV. We confirmed that the crystallinity was degraded, and carbon-related contamination was detected at the surface and edge of the aperture hole of the Si electron lens after electron bombardment for 24 h. Carbon-related contamination on the surface of the Si electron lens was verified by analyzing the Raman spectra of the carbon-deposited Si substrate using DC sputtering and a carbon rod sample. We report the crystallinity and the origin of the carbon-related contamination of electron Si lenses after electron beam bombardment by non-destructive Raman scattering and XPS analysis methods.

## 1. Introduction

Microcolumns, which are stacked structures containing an electron emission source, an electron lens (source lens and einzel lens), and a deflector, have many advantages owing to their size (1/100) compared to existing electron columns [1,2,3]. In particular, the advanced micro electro-mechanical system (MEMS) process enables the development of a miniaturized structure and the mass production of microcolumns, and it can create a multi-beam structure using n × n array microcolumns [4].

Accelerated electrons, through the electron emission source, pass through the aperture hole of the first electrode, which is the extractor, thereby causing collisions around the aperture hole. Consequently, various types of defects are formed on the silicon surface. Carbon is simultaneously deposited around the aperture hole to generate contamination, which occurs when the source generated during sample measurement in scanning electron microscope (SEM) analysis is deposited onto the sample surface. Carbon-related contamination can be generated from carbon molecules generated during the preparation of the SEM sample, the use of carbon tape, the vacuum level of the chamber of the SEM system, and the use of vacuum grease. Additionally, carbon-related contamination is generated based on external environmental factors [5,6,7]. As the sources of carbon-related contamination generated by accelerated electrons degrade the precision of sample analysis, many researchers have attempted to minimize contamination [8]. This phenomenon has been incorporated into the electron beam-induced deposition process and further utilized as a fine compound deposition technology [9].

All components of the fabricated silicon electron lens were assembled in the microcolumn between the silicon electron lens gap, which is very narrow so that a high electric field can be generated between the lenses. The emitted electrons continuously collided around the aperture hole owing to the high electric field of the electronic lens and the crystallinity of silicon degrades, thereby shortening their lifetime. Several methods, such as scanning electron microscopy (SEM) and transmission electron microscopy (TEM), have been proposed for the structural analysis of the sample surface. However, these are destructive methods for the analysis of raw samples. Pre-treatment is time-consuming and very expensive for measuring the sample. As carbon-related contamination is deposited near the aperture hole during electron beam irradiation, it adversely affects the final product. Therefore, monitoring and analyzing the crystallographic changes in the silicon electron lens is challenging. In this study, the generated carbon and the change in silicon crystallinity caused by the collision of electrons with several acceleration voltages around the aperture hole of the electron lens were verified using Raman scattering analysis. Furthermore, the carbon deposited on a bare silicon wafer with a DC sputter, carbon rod, and carbon isotope were compared using Raman scattering to analyze the effects of electron bombardment. The Raman scattering method is non-destructive and has the advantage of a relatively short analysis time because it can obtain the electron structure in any phase material, chemical composition, and structural information without pretreatment or damage to the samples [10]. The non-destructive Raman scattering method can positively influence an industrial economy by predicting the lifetime of silicon electron lens using continuous electron bombardment.

## 2. Results

### 2.1. Raman Spectra of MEMS Si Electron Lens Irradiated with Electron Beam

A Si electron lens was manufactured using the MEMS process. Silicon wafer with a thickness of 400 μm was coated with a photoresist (PR) and the aperture hole was patterned. Deep reactive ion etching (DRIE) was used to develop the aperture hole. The silicon oxide film layer was formed by thermal oxidation to protect the aperture hole. PR patterning, the oxide film, and etching were performed on the opposite side, and the PR was removed. To prepare a membrane layer, the opposite side was etched until the oxide film fabricated on the aperture hole was exposed. The oxide film was removed, resulting in a silicon membrane layer with a thickness of at least 20 μm and a diameter of 10 μm to 200 μm, depending on the pattern type. The Si electron lens sample was fabricated using the MEMS process. After the electron beam was irradiated with an acceleration voltage of 0 eV, 20 keV, and 30 keV around the aperture hole of the etched Si electron lens sample for 24 h, the surface of the silicon near the aperture hole was irradiated using Raman scattering.

Single-crystal silicon is a semiconductor with a diamond structure. Typically, silicon (100) can be easily used to form oxides in semiconductor devices and MEMS processes. The first-order Raman spectrum (Γ point, k~0) of stress-free single-crystal silicon at room temperature (300 K) is primarily observed at 520.3 cm^−1^ in Raman scattering measurement [11]. The peak around 520 cm^−1^ (longitudinal optic: LO) that we addressed is the peak of the first-order phonon, and the band around ~1000 cm^−1^ is the second-order phonon band of silicon. As the concentration of crystal defects inside the silicon crystal increases, the intensity of the Raman spectrum decreases, the full width at half maximum (FWHM) of the spectrum increases, and the shape of the Raman spectrum changes asymmetrically [12].

**Figure 1:**
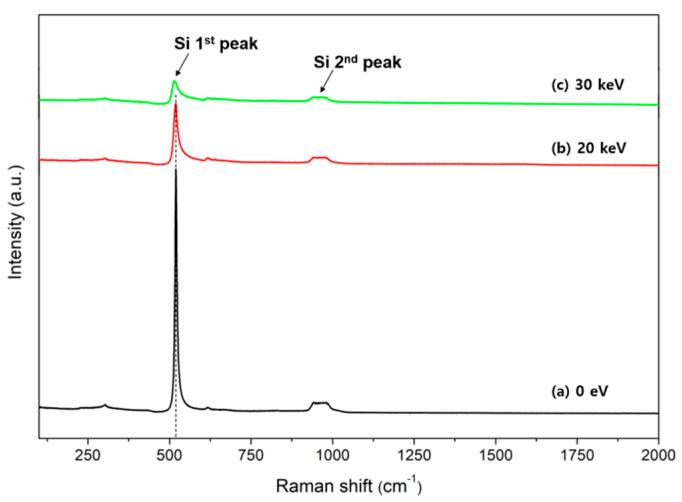
Raman spectra after the irradiation of electron beams with acceleration voltages of (**a**) 0 eV, (**b**) 20 keV, and (**c**) 30 keV for 24 h around the aperture hole of the Si electron lens fabricated using the micro electro-mechanical system (MEMS) process.

Figure 1 shows the Raman spectra of the Si electron lens samples (fabricated by the MEMS process) irradiated with electron beams with acceleration voltages of 0 eV, 20 keV, and 30 keV around the aperture hole for 24 h. The Raman spectrum of the 0 eV acceleration voltage sample shown in Figure 1a is the same as the stress-free single-crystal silicon. As shown in Figure 1b,c, as the electron beam energy increased to 20 keV and 30 keV, the peak intensity at 520 cm^−1^ decreased, and the FWHM increased.

**Figure 2:**
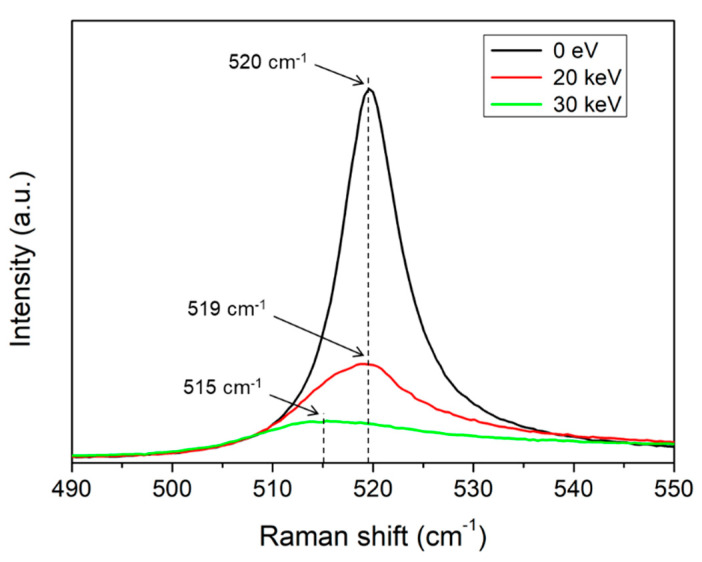
Raman spectrum waveband (490 cm^−1^–550 cm^−1^) after irradiating electron beams with acceleration voltages of 0 eV, 20 keV, and 30 keV around the aperture hole of a Si electron lens fabricated using the MEMS process for 24 h.

Figure 2 shows the Raman spectrum of silicon corresponding to the range between 490 cm^−1^ and 550 cm^−1^, as shown in Figure 1. The average center wavenumbers obtained by measuring the four points around the aperture hole of the Si electron lens were 520 cm^−1^, 519 cm^−1^, and 515 cm^−1^, respectively. The spectrum was shifted to a lower wavenumber as the acceleration voltage increased. This indicates that as the acceleration voltage increases, electron beam bombardment is applied intensively to the aperture hole, thereby weakening the bonding of silicon atoms in the Si electron lens. As the acceleration voltages of 0 eV, 20 keV, and 30 keV increased, the average FWHM increased to 9 cm^−1^, 19 cm^−1^, and 29 cm^−1^, respectively. This indicates the formation of the amorphous phase of the Si electron lens after irradiation with electron beams.

**Figure 3:**
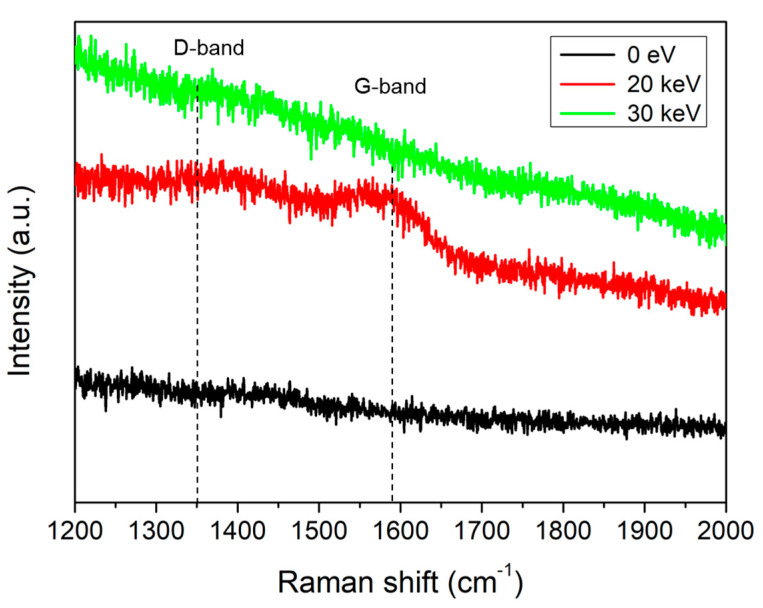
Raman spectrum waveband (1200 cm^−1^ to 2000 cm^−1^) after irradiating electron beams with acceleration voltages of 0 eV, 20 keV, and 30 keV around the aperture hole of a Si electron lens fabricated using the MEMS process for 24 h.

Figure 3 shows the Raman spectra from 1200 cm^−1^ to 2000 cm^−1^, as shown in Figure 1. The carbon-related bands, D-band and G-band, were weakly observed in the Si electron lens sample irradiated to an electron beam acceleration voltage of 20 keV. This result indicates that the electron beam is continuously bombarded around the aperture hole, resulting in the degradation of the crystallinity of the silicon near the aperture hole. The carbon-related contamination was simultaneously deposited near the aperture hole.

Three Raman bands were observed in highly oriented pyrolytic graphite (HOPG). The D-band and G-band were observed at ~1350 cm^−1^ and ~1590 cm^−1^, respectively. The D-band is the peak associated with a defect. The G-band is a common peak in graphite-related substances [13].

The carbon-related peak was weakly observed in the Si electron lens sample irradiated to an electron beam acceleration voltage of 20 keV. However, the D-band and the G-band disappeared in the sample with an acceleration voltage of 30 keV. This indicated that the bond between the deposited carbon atoms and Si weaken due to continuous bombardment irradiated by a relatively strong electron beam.

### 2.2. Raman Spectra of Carbon Rod and Carbon Deposited Si

The carbon-related band additionally observed in the Si electron lens irradiated with an acceleration voltage of 20 keV electron beam for 24 h was confirmed by analyzing the Raman spectra based on the sample deposited by sputtering carbon on the surface of the carbon rod (Cressington, Watford, UK) and the Si electron lens. In this case, carbon allotropes, such as carbon rods and carbon nanotubes (CNTs), as well as phonon peaks corresponding to the D-band, G-band, and 2D-band, were identified and commonly observed [14,15,16,17,18,19,20,21].

**Figure 4:**
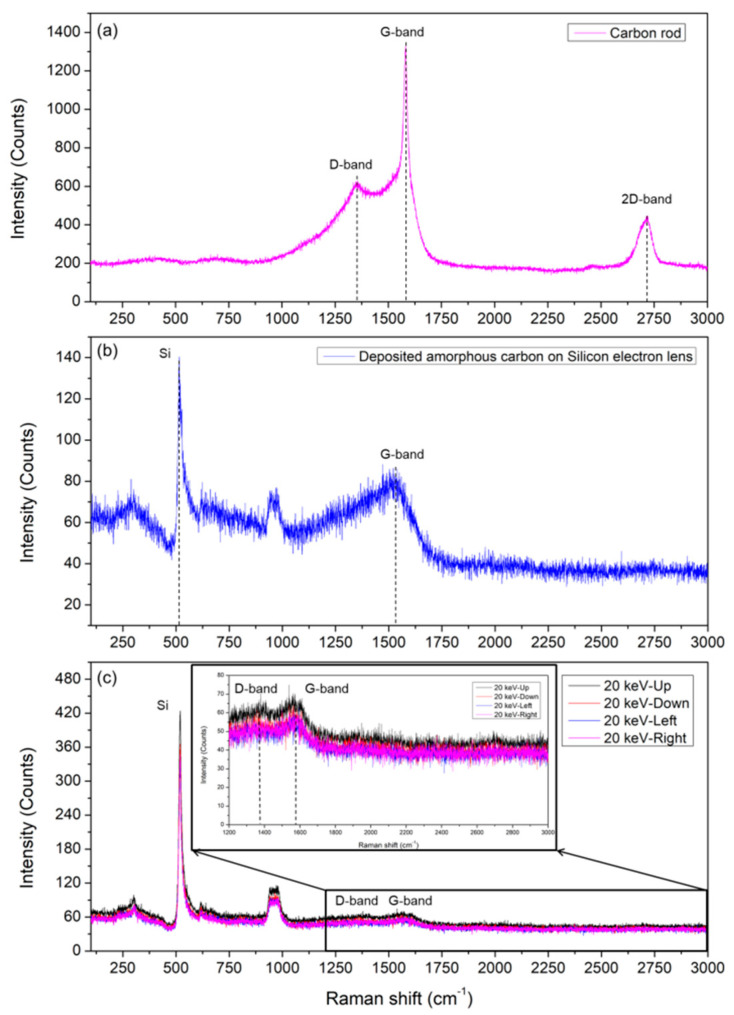
Raman spectra of carbon-related materials. (**a**) Carbon rod; (**b**) amorphous carbon deposited by D.C sputtering on a Si electron lens; (**c**) Si electron lens irradiated with 20 keV (inset: 1200 cm^−1^–3000 cm^−1^).

Figure 4 shows the Raman spectra of the carbon-related material prepared using various methods. Figure 4a shows the Raman spectrum of a typical carbon rod. The G-band, D-band, and 2D-band were observed at 1581 cm^−1^, 1355 cm^−1^, and 2718 cm^−1^, respectively. Figure 4b shows the Raman spectrum of the carbon deposited Si electron lens using a DC sputter. The thickness of the deposited carbon layer was approximately 750 nm (KLA Tencor, Milpitas, CA, USA).

The G-band, shown in Figure 4b, was observed at approximately 1500 cm^−1^, which was significantly shifted to the low-frequency side compared to the G-band of the carbon rod shown in Figure 4a. This indicates the weak bonding of carbon atoms on the Si surface. A band near 520 cm^−1^ for the LO phonon of silicon was weakly observed. Compared to the center wave of stress-free single-crystal silicon at 520 cm^−1^, this band is near 516 cm^−1^ at a low wavenumber, which is shifted by approximately 4 cm^−1^. This result suggests that the silicon crystal is subject to relative tensile stress induced by the carbon layer deposited on the Si electron lens substrate.

The probing depth (δ = 1/2α) of the silicon single-crystal for the 532 nm excitation light source is determined by the absorption coefficient (α) of the silicon crystal for the excitation light source, which is approximately 800 nm from the surface [11]. Thus, the observed Raman spectrum contains the average information of all phonons ranging from the carbon layer deposited onto the surface of the Si electron lens to the surface of the Si electron lens below.

Figure 4c shows the Raman spectrum measured at four points: the top, bottom, left, and right of the center of the aperture hole of the Si electron lens irradiated with an electron beam of 20 keV for 24 h. At the four measured points, a sharp peak for the LO phonon of silicon (520 cm^−1^) and weak peaks for the D-band and G-band (1372 cm^−1^ and 1567 cm^−1^) were observed. As previously described, this result indicates that the electron beam is focused on the aperture hole of the Si electron lens, and the significant degradation of the crystallinity of Si occurs near the aperture edge with a relatively high surface area. The degradation creates an energetically unstable area around the edges, indicating that carbon-related contamination around the site is intensively adsorbed. As a result, the amorphization of the silicon crystal proceeds owing to the degradation of the crystallinity as continuous damage is applied around the aperture edge.

### 2.3. XPS Analysis of MEMS Si Electron Lens Irradiated with Electron Beam

**Figure 5:**
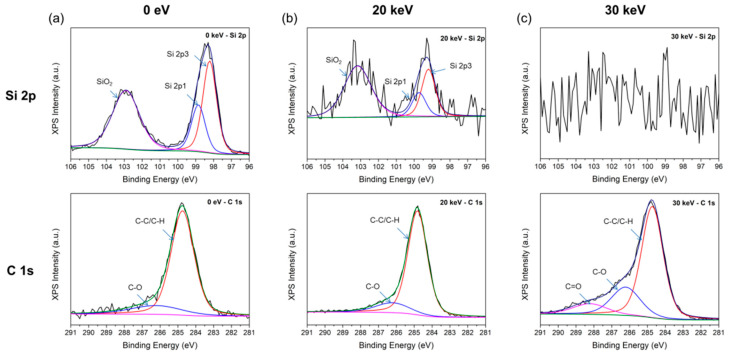
XPS spectra of Si 2p and C 1s states MEMS Si electron lens irradiated with electron beam of (**a**) 0 eV; (**b**) 20 keV; (**c**) 30 keV. (all C-C peaks were calibrated at 284.8 eV in the C 1s XPS spectra).

X-ray photoelectron spectroscopy (XPS) is a non-destructive analysis method that is widely used for the analysis of materials [22]. The origin of the carbon was analyzed using XPS measurements. The electron beams were irradiated with acceleration voltages of 0 eV, 20 keV, and 30 keV to three Si electron lenses. Monochromated Al-Kα (*hν* = 1486.6 eV) was used as the X-ray source. The X-ray probing depth was approximately 10 nm from the sample surface. Figure 5 shows the XPS spectra of the Si 2p and C 1s states according to acceleration voltages of 0 eV, 20 keV, and 30 keV (all C-C peaks were calibrated at 284.8 eV in the C 1s XPS spectra).

In the case of the Si 2p state, SiO_2_, Si 2p1, and Si 2p3 bands in the 0 eV sample (Figure 5a) are clearly observed, while the band intensity relatively weakened in the 20 keV sample. All Si-related bands in the 30 keV sample finally disappeared. These results imply that the crystallinity of the Si electron lens of the 30 keV sample is defective, as shown in Figure 2 (Raman spectra).

In the case of state C, the amount of carbon-related contamination around the aperture hole increased as the acceleration voltage increased to 30 keV due to the shortening of the probing depth by carbon.

Table 1 lists the elemental composition of the Si electron lens samples. XPS analysis showed that the silicon contents of the 0 eV, 20 keV, and 30 keV samples were 31.4%, 1.5%, and 0%, respectively.

The carbon contents of the 0 eV, 20 keV, and 30 keV samples were 36.7%, 88.9%, and 78.0%, respectively. The C-C bond and C-O bond contents at C 1s states of 0 eV, 20 keV, and 30 keV were 24.4%, 76.2%, and 52.4% and 4.5%, 12.5%, and 17.7%, respectively. The C=O and C-O bond contents in the C 1s band at 30 keV (7.9% and 17.7%, respectively) were higher than those of the 20 keV sample (0% and 12.5%, respectively). The C-C bond content of the 20 keV sample was higher than that of the 30 keV sample. These results are in accordance with the appearance of a carbon-related D-band and G-band in the 20 keV sample during Raman measurement.

## 3. Discussion

Most electrons emitted from the electron emission source, comprising the microcolumn, pass through the aperture hole of the Si electron lens. The electrons that fail to pass through hit the area around the aperture hole, causing electron bombardment. Therefore, electron bombardment can affect the lifetime of the electronic lens inside the microcolumn. This study analyzed impacts on the crystallinity of Si electron lenses, which were fabricated using the MEMS process, by irradiation to an electron beam for 24 h with various acceleration voltages using the Raman spectrum. We observed that the crystallinity of silicon deteriorated around the aperture hole of the electron lens due to the bombardment of a continuous electron beam of approximately 20 keV, where carbon atoms were deposited on the surface. Moreover, the silicon crystallinity severely deteriorated on the surface of the Si electron lens bombarded by an electron beam of 30 keV or more. These results confirm that the crystallinity of silicon is degraded by continuous bombardment of electron beams around the aperture hole of the MEMS Si electron lens. Furthermore, a change observed in the Raman spectrum indicates that the amorphization of the silicon crystal occurred due to deterioration. The overall results suggest that surrounding carbon-related contamination is deposited around the atoms near the edge of the relatively high-surface-area aperture hole. XPS analysis indicated that the carbon content and carbon-related bonds in the C state increased with increasing acceleration voltage. This trend was in accordance with the Raman spectra analysis. In the future, additional studies will be conducted to suppress or reduce the progression of the carbon-related contamination of MEMS Si electron lenses.

## 4. Materials and Methods

### 4.1. Microcolumn Lens Electrode Fabrication Process

Si electron lenses used in microcolumns use a piece of silicon, which is fabricated by processing a silicon wafer as an electrode. Silicon wafers are highly doped with boron to improve electrode efficiency and serve as an etch-stop.

**Figure 6:**
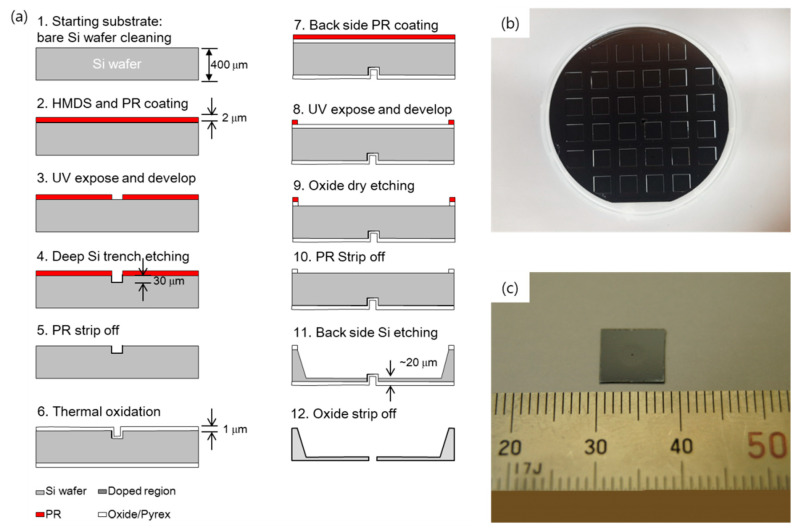
(**a**) Schematic diagram of the fabrication process of a Si electron lens; (**b**,**c**) Si electron lens wafer fabricated using the MEMS process.

Figure 6 shows the fabrication process of the Si electron lens, the processed Si wafer, and a piece of the Si electron lens. Figure 6a shows the entire manufacturing process, which is divided into two parts: forming the aperture of the electrode and membrane of the electrode.

The 12 steps of the fabrication process are shown in Figure 6a. First, hexamethyldisilazane (HMDS) and a photoresist (PR) were coated during processes 1 to 3 onto a silicon wafer with a thickness of approximately 400 μm, and the aperture shape was patterned. The aperture was prepared in various sizes ranging from 1 μm to 200 μm, based on the role of each part of the electronic lens. Next, using DRIE, an aperture hole of approximately 30 μm or more was prepared in the silicon wafer according to the PR pattern. To fabricate a hole with a uniform shape, the hole depth must be greater than the membrane thickness of the electrode to be fabricated. In the next process, the PR was removed, and a silicon oxide layer was formed by thermal oxidation to protect the aperture and pattern formation for membrane development. Next, in processes 7 to 11, the PR was coated onto the opposite side of the silicon wafer where the aperture was formed, and the PR was removed after PR patterning. Oxide film etching was then performed. Finally, if the oxide film was removed in process 12 to form a silicon membrane layer, after etching the opposite side until the oxide film prepared on the aperture hole was exposed (process 11), a silicon membrane layer with a thickness of at least 20 μm and a diameter of 10 μm to 200 μm was fabricated based on the PR pattern.

Figure 6b shows the 32 electron lenses obtained through the MEMS process on a 4-inch Si wafer. As shown in Figure 6c, the length of the electronic lens was approximately 10 mm × 10 mm.

### 4.2. Raman Spectroscopy

In this experiment, we obtained measurements at room temperature using a Raman spectrometer (Horiba, Kyoto, Japan). A solid-state laser with a wavelength of 532 nm and an output of 500 mW was utilized as an excitation light source. The Raman scattering measurement method uses a back-scattering configuration, in which a laser is irradiated onto the crystal surface (in the crystal axis direction) of the silicon and the scattered light is focused back on the detector. The size of the laser focal point focused on the sample was approximately 100 μm in diameter, and a 50× objective lens with a numerical aperture (NA) of 0.75 was used. The detector applied a charge-coupled device (CCD) and diffraction grating of 1800 grooves/mm. In this case, the wavenumber resolution was calculated as 0.4 cm^−1^. The Raman spectrum measurement range was 100 cm^−1^ to 3000 cm^−1^.

### 4.3. Electron Beam Irradiation to Silicon Electron Lens through EPMA

Si electron lenses irradiated with electron beams were fabricated by scanning electron beams for 24 h at 20 keV and 30 keV, using an electron probe micro-analyzer (EPMA) (JEOL, Japan). The scan region was a 1 mm^2^ area centered on the aperture hole of the silicon electron lens, with a probe current of 1 μA emitted from the tungsten field emitter and irradiated to the sample. Raman analysis was conducted using the same method as for the prepared sample, and the results were compared with those of the non-irradiated sample.

**Figure 7:**
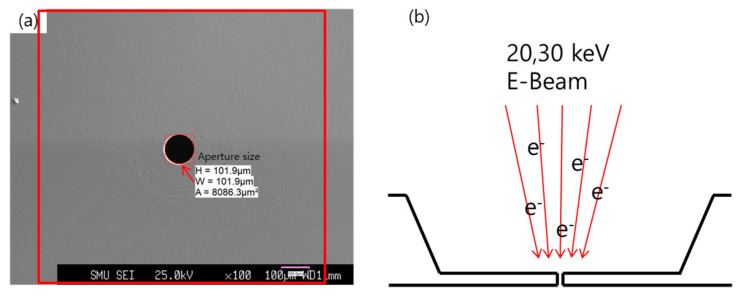
(**a**) SEM image showing a ×100 magnification of the aperture hole of an electron lens (red box: 1 mm × 1 mm area irradiated with an electron beam). (**b**) Cross-sectional schematic diagram of an electron beam irradiated onto the aperture hole of the Si electron lens.

Figure 7a shows the SEM image (red box: 1 mm^2^ area irradiated with an electron beam) of the aperture hole of the Si electron lens magnified ×100. The size of the aperture hole was measured using image analysis software (WaferMasters, Dublin, CA, USA). Both the horizontal and vertical diameters were 101.9 μm, and the aperture hole of the area was 8086 μm^2^ [23,24]. Figure 7b shows a cross-sectional schematic of the electron beam irradiated onto the aperture hole of the Si electron lens.

## 5. Conclusions

This study evaluated irradiated electron beams of various acceleration voltages near the aperture hole of a Si electron lens fabricated using the MEMS process and further analyzed the crystallinity change by a non-destructive method using Raman scattering. According to the results, as the acceleration voltage of the electron beam increased, the intensity of the LO phonon peak of silicon at 520 cm^−1^ decreased and the FWHM increased, corresponding to a shift to a low-wave band. These results indicate that the higher the acceleration voltage, the greater the number of electron bombardments near the aperture edge with a high surface area, resulting in defective silicon crystals. Furthermore, the results show that the amorphization of silicon crystals progresses due to deterioration. In addition, the results confirmed that the surrounding carbon-related contamination was adsorbed near the aperture edge of the Si electron lens with a relatively high surface area, owing to the electron bombardment caused by a relatively high acceleration voltage. The method utilized in this study will have a positive economic effect on the semiconductor industry owing to the use of Raman scattering and XPS analysis for predicting the lifetime of Si electron lenses fabricated by MEMS. An additional advantage is the long-term and non-destructive analysis of the deposition of carbon-related contamination onto the surface of Si electron lenses under a specific acceleration voltage.

## Figures and Tables

**Table 1:** Elemental composition (%) of the Si electron lens samples.

XPS Sample	Elemental Composition (%)				C 1s			Si 2p			O 1s			Na 1s
	Si	C	O	Na	C-C	C-O	C=O	SiO_2_	Si 2p1	Si 2p3	C-O	C=O	SiO_2_	Na 1s
0 eV	31.4	36.7	29.4	2.5	24.4	4.5	-	14.3	0	17.6	5.2	-	31.6	4.1
20 keV	1.5	88.9	9.6	-	76.2	12.5	-	0.9	0	0.6	1.7	-	8.1	-
30 keV	-	78.0	22.0	-	52.4	17.7	7.9	-	-	-	11.6	10.4	-	-

## Data Availability

Not applicable.

## Sample Availability

Samples of the compounds are not available from the authors.

## References

[1] Oh T.S., Kim D.W., Ahn S.J.A., Kim Y.C., Kim H.S., Ahn S.J. (2008). Optimization of Electrostatic Lens Systems for Low-Energy Scanning Microcolumn Applications. J. Vac. Sci. Technol. A.

[2] Kim H.S., Ahn S.J., Kim D.W., Oh T.S., Ahn S.J. (2008). Efficient Electron Beam Condensing for Low-Energy Micro-Column Lithography. Microelectron. J..

[3] Kratschmer E., Kim H.S., Thomson M.G.R., Lee K.Y., Rishton S.A., Yu M.L., Zolgharnain S., Hussey B.W., Chang T.H.P. (1996). Experimental evaluation of a 20 × 20 mm footprint microcolumn. J. Vac. Sci. Technol. A.

[4] Lee Y.B., Kim H.W., Kim D.W., Ahn S.J., Kim H.S. (2020). The Design and Fabrication of a Wafer-Scale Microlens for Multiple Microcolumns. J. Multifunct. Mater. Photosci..

[5] Reimer L. (1993). Transmission Electron Microscopy-Physics of Image Formation and Microanalysis.

[6] Reimer L., Wächter M. (1978). Contribution to the Contamination Problem in Transmission Electron Microscopy. Ultramicroscopy.

[7] Lau D., Hughes A.E., Muster T.H., Davis T.J., Glenn A.M. (2010). Electron-Beam-Induced Carbon Contamination on Silicon: Characterization Using Raman Spectroscopy and Atomic Force Microscopy. Microsc. Microanal..

[8] Kim Y.M., Choi J.H., Song K., Kim Y.S., Kim Y.J. (2009). Experimentally Minimized Contaminative Condition of Carbonaceous Artifacts in Transmission Electron Microscope, Korean. J. Microsc..

[9] Wei X.L., Liu Y., Chen Q., Peng L.M. (2008). Controlling Electron-Beam-Induced Carbon Deposition on Carbon Nanotubes by Joule Heating. Nanotechnology.

[10] No J.H., Lee T.K. (2017). Rapid Bacterial Identification Using Raman Spectroscopy. Korean J. Microbiol..

[11] Yoo W.S., Kim J.H., Han S.M. (2014). Multiwavelength Raman Characterization of Silicon Stress near Through-Silicon vias and Its Inline Monitoring Applications. J. Micro/Nanolithogr. MEMS MOEMS.

[12] Adu K.W., Williams M.D., Reber M., Jayasingha R., Gutierrez H.R., Sumanasekera G.U. (2012). Probing Phonos in Nonpolar Semiconducting Nanowires with Raman Spectroscopy. J. Nanotechnol..

[13] Thomsen C., Reich S. (2000). Double Resonant Raman Scattering in Graphite. Phys. Rev. Lett..

[14] Wang Y., Alsmeyer D.C., McCreery R.L. (1990). Raman Spectroscopy of Carbon Materials: Structural Basis of ObservedSpectra. Chem. Mater..

[15] Vidano R., Fischbach D.B. (1978). New Lines in the Raman Spectra of Carbons and Graphite. J. Am. Ceram. Soc..

[16] Nemanich R.J., Solin S.A. (1979). First- and Second-Order Raman Scattering from Finite-Size Crystals of Graphite. Phys. Rev. B.

[17] Nemanich R.J., Solin S.A. (1977). Observation of an Anomolously Sharp Feature in the 2nd Order Raman Spectrum of Graphite. Solid State Commun..

[18] Tuinstra F., Koenig J.L. (1970). Raman Spectrum of Graphite. J. Chem. Phys..

[19] Dresselhaus M.S., Dresselhaus G., Saito R., Jorio A. (2005). Raman Spectroscopy of Carbon Nanotubes. Phys. Rep..

[20] Sapirstein V., Yang Y., Zhou W.L., Lashmore D., Schauer M. (2007). Raman Analysis of Single-Walled Carbon Nanotube (SWCNT) Films. Spectroscopy.

[21] Kim H.G., Kim Y.S., Kwac L.K., Shin H.K. (2020). Characterization of Activated Carbon Paper Electrodes Prepared by Rice Husk-Isolated Cellulose Fibers for Supercapacitor Applications. Molecules.

[22] David S.J., Supriya S.K., Nitesh M., Michael A.V., Andrew E.D., Mark H.E., Matthew R.L. (2013). Silicon (100)/SiO_2_ by XPS. Surf. Sci. Spectra.

[23] Yoo W.S., Kang K.T., Kim J.G., Ishigaki T. Image-Based Dimensional Analysis for Semiconductor and MEMS Structures. Proceedings of the 2019 International Conference on Frontiers of Characterization and Metrology for Nanoelectronics (FCMN).

[24] Yoo W.S., Ishigaki T., Kang K.T. Image Processing Software Assisted Quantitative Analysis of Various Digital Images in Process Monitoring, Process Control and Material Characterization. Proceedings of the 2017 International Conference on Frontiers of Characterization and Metrology for Nanoelectronics (FCMN).

